# Melanoma Cells Revive an Embryonic Transcriptional Network to Dictate Phenotypic Heterogeneity

**DOI:** 10.3389/fonc.2014.00352

**Published:** 2014-12-09

**Authors:** Niels Vandamme, Geert Berx

**Affiliations:** ^1^Unit of Molecular and Cellular Oncology, Inflammation Research Center, VIB, Ghent, Belgium; ^2^Department of Biomedical Molecular Biology, Ghent University, Ghent, Belgium

**Keywords:** melanoma, EMT, phenotype-switching, drug-resistance, ZEB1, ZEB2, SLUG, MITF

## Abstract

Compared to the overwhelming amount of literature describing how epithelial-to-mesenchymal transition (EMT)-inducing transcription factors orchestrate cellular plasticity in embryogenesis and epithelial cells, the functions of these factors in non-epithelial contexts, such as melanoma, are less clear. Melanoma is an aggressive tumor arising from melanocytes, endowed with unique features of cellular plasticity. The reversible phenotype-switching between differentiated and invasive phenotypes is increasingly appreciated as a mechanism accounting for heterogeneity in melanoma and is driven by oncogenic signaling and environmental cues. This phenotypic switch is coupled with an intriguing and somewhat counterintuitive signaling switch of EMT-inducing transcription factors. In contrast to carcinomas, different EMT-inducing transcription factors have antagonizing effects in melanoma. Balancing between these different EMT transcription factors is likely the key to successful metastatic spread of melanoma.

## Introduction

Malignant melanoma is an aggressive skin cancer arising from the melanocyte lineage. Despite extensive research efforts on the molecular aspects of malignant melanoma, no effective therapies exist and nearly all patients with advanced stage melanomas die due to its distant metastases after 6–10 months (1). Melanoma is one of the most difficult cancers to treat successfully because it disseminates very early after the development of the initial lesion. Melanomas are notorious for development of drug-resistance and many patients are refractory to single-agent therapies including newly developed drugs such as vemurafenib (2). Surgical excision after early detection still remains the most effective therapy, but it has only a limited impact on disseminated melanoma. Accumulating evidence indicates intratumoral heterogeneity as a key determinant of the emergence therapy-resistant cells, which lead to treatment failure and tumor recurrence. Single-agent drugs usually target one subpopulation of melanoma cells while other subpopulations survive, repopulate a tumor or spread to distant sites. Characterization of melanoma subpopulations, and in particularly the signaling events that regulate phenotypic heterogeneity, might lead to the development of combination therapies targeting multiple subpopulations of tumor cells, which could favor long-term remissions. Moreover, classifying the tumor phenotypes could help identify patients who might benefit from existing and new therapeutics.

Phenotypic heterogeneity arises among melanoma cells within the tumor as a consequence of (epi)genetic mutations, micro-environmental cues, and reversible changes within the cells. Over the last few years, several research groups studied distinct subpopulations in melanoma and framed their observations in different conceptual models to explain the phenotypic and functional diversity of melanoma cells. In all of these models, signaling events involved in melanoma cellular plasticity are intertwined with transcription factors known to induce epithelial-to-mesenchymal transitions (EMT) in epithelial context. EMT is the process in which epithelial cells lose their epithelial characteristics, gain mesenchymal features, and become motile. Different EMT-controlling transcription factors such as SNAIL, SLUG, ZEB1, and ZEB2 have been identified and characterized thoroughly as important drivers of EMT (3). Reactivation of EMT-inducing transcription factors in epithelial cells promotes oncogenic transformation and dissemination EMT in carcinoma cells is accompanied by an increase in angiogenesis, invasiveness, and drug-resistance, leading to a more aggressive tumor type. Compared to the overwhelming amount of literature describing how these EMT transcription factors orchestrate cellular plasticity in embryogenesis and epithelial cells, the functions of these factors in non-epithelial contexts, such as melanoma, are not clear. This mini review highlights the models that account for phenotypic and functional heterogeneity of melanoma and how EMT-inducing transcription factors fit into the molecular circuitries relevant for these different models.

## Clonal Evolution vs. Cancer-Stem Cell Model

Genetic instability that drives clonal tumor evolution is the first model to account for tumor heterogeneity and diversity described in 1976. This model states that (epi)genetic changes occur over time in all tumor cells, and that cells gaining a selective advantage will generate clones that out-compete other clones (4). Stepwise natural selection of the fittest and most aggressive cells would facilitate tumor progression in a linear stochastic manner.

An alternative explanation for the intratumoral heterogeneity in melanoma is the cancer-stem cell model. According to this model, a tumor is composed of cell populations in various states of differentiation, with a large subgroup of rapidly dividing and differentiated tumor cells and another small subgroup with a quiescent stem cell-like phenotype responsible for tumor spread and growth (5). Consequently, slow-cycling cancer-stem cells may evade chemotherapeutics that usually target replicating cancer cells. Several research groups have proposed that transcription factors regulating EMT also generate tumor-initiating cancer-stem cell characteristics (6–9). Evidence supporting the concept of EMT-inducing transcription factors conferring cancer-stem cell features to tumor cells comes mainly from epithelial-derived cancers, whereas, the link between melanoma stem cells and EMT activators is less clear. Typical hallmarks of malignancy in melanoma cancer-stem cells have been associated with EMT-inducing transcription factors as discussed below, but due to controversy surrounding certain melanoma cancer-stem cell markers, a clear correlation between these transcription factors and specific markers is lacking. ZEB1 downregulation in B16-F10 melanoma cells and in its presumed cancer-stem cell CD133^+^ CD44^+^ subpopulation reduced their tumor growth *in vivo*, yet it has not been shown whether lack of ZEB1 effectively depletes the CD133^+^ CD44^+^ fraction in B16-F10 tumors as such (10).

## Phenotype-Switching and EMT Modulators

Some cancer types display a clear hierarchy of tumorigenic and non-tumorigenic cells, but this hierarchy is accompanied both by irreversible genetic instability as well as by reversible phenotypic changes. A third model featuring phenotypic diversity deals with these reversible phenotypic changes in melanoma cells referred to as phenotype-switching, independently of hierarchical organization. This model emphasizes reversible switching between different phenotypes of proliferative and invasive potentials. Besides cell-intrinsic factors regulating proliferation and migration, micro-environmental cues such as nutrients, oxygen, cytokines, and growth factors affect this reversible switch. Melanoma progression does not rely solely on irreversible clonal or lineage-driven remodeling, but can be driven by reversible and functional reprograming of signaling routes. This functional reprograming is centered on microphthalmia-associated transcription factor (MITF), the master regulator of melanocyte differentiation, and pigmentation genes. MITF is a lineage-specific oncogene that is frequently amplified or overexpressed in human melanomas (11). The role of MITF in melanoma is controversial, as MITF levels are subjected to a tight regulation and different levels of MITF exert different effects in melanomagenesis (12). Hoek and Goding (13) defined two molecular signatures corresponding to two opposing phenotypes: the G1-arrested invasive phenotype with cancer-stem cell properties and the MITF-driven non-invasive proliferative and differentiated phenotype. In this concept, the phenotype switch can be regulated by different signaling routes and MITF acts as a “rheostat” that determines the various plasticity states. MITF-depleted cells have a more stem cell-like phenotype, increased plasticity, and reduced proliferation, which collectively favor tumor progression, whereas high levels of MITF promote proliferation and differentiation (14). Anti-senescence signaling is likely to take place in these cells because long-term depletion of MITF in melanoma cells triggers a senescence program associated with sustained growth arrest (15). The proposed model matches the observations of Pinner et al. of observed Brn2 and melanin (hallmarks of invasion and differentiation, respectively), which are present in a mutually exclusive manner in melanoma cells as they invade and disseminate (16). Over the last years, several research groups have reported reversible mechanisms of invasion that support the phenotype-switching model (17–21). The strict dichotomy between proliferation and invasion can be nuanced, as it has been reported that proliferation and invasion are not always mutually exclusive states in melanoma (22). These findings coincide with the observation that there is no clear correlation between proliferation rate and tumorigenic cell frequency in human melanomas (23).

**Figure 1:**
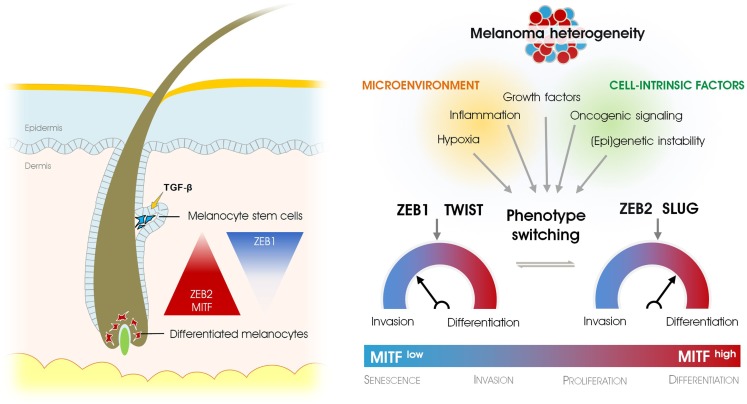
**Epithelial-to-mesenchymal transition-inducing transcription factors in physiological and pathological development of the melanocyte lineage**. EMT-inducing transcription factors regulate stemness and differentiation in melanocytes (left), whereas they determine the oscillation between differentiated vs. invasive cancer cells in melanoma (right). Phenotype-switching that accounts for melanoma heterogeneity depends on a signaling switch of different EMT-inducing transcription factors and is regulated by microenvironmental cues, (epi)genetic instability, and oncogenic signaling.

The phenotypic switch is coupled with an intriguing and somewhat counterintuitive signaling switch of EMT-inducing transcription factors. We and others have shown how EMT-inducing transcription factors are crucial for acquiring an invasive or differentiated state subjected to respectively low or high levels of MITF (24, 25). We found that the transcription factor ZEB2 is strongly expressed in migrating melanoblasts in the embryo and that this expression persists in differentiated melanocytes in the hair follicle where it is maintained throughout adult life as an important gatekeeper of melanocyte differentiation. Although melanin pigment and differentiation markers such as MITF and tyrosinase are lost when ZEB2 is specifically deleted in the melanocytic lineage, undifferentiated non-pigmented melanocytes can remain in the hair follicle. Loss of ZEB2 is associated with loss of MITF and subsequent dedifferentiation. In addition, an increase in ZEB1 expression has been observed. The paradoxical expression of ZEB2 in normal melanocytes also holds true for SLUG, which is strongly expressed in neural crest-delaminated melanoblasts, melanocytes, and benign nevi (26). These findings may invalidate the oncogenic potential of SLUG and ZEB2 in the melanocytic lineage because both proteins play important roles in the differentiation of melanocytes. Indeed, both Caramel et al. (24) and Denecker et al. (25) have shown that expression of SLUG and ZEB2 are positive prognostic factors for melanoma patients. Besides the effects of ZEB2 expression on MITF and cellular plasticity, ZEB2 might also have a tumor-suppressive role due to its modulation of PTEN in a complex RNA-regulatory network in melanoma (27). Based on human melanoma samples, a switch from ZEB2/SLUG to ZEB1/TWIST expression indicates malignant progression. ZEB1 and TWIST induce a molecular signature associated with a TGF-β driven profile of migration and invasion and downregulation of MITF (24). Moreover, this signature can be reversed by ectopic expression of ZEB2 or SLUG. ZEB1 may drive a failsafe program for a melanoma cell to overcome the senescent state, exemplifying why ZEB1 expression in human melanoma is inversely correlated with MITF status. The recent work on EMT-inducing transcription factors in melanoma leads to the idea that melanoma cells cycle between a differentiated and state (high levels of ZEB2 and SLUG) and a oncogenic invasive phenotype (high levels ZEB1 and TWIST). This reversible reprograming of EMT-TF is driven by increased MAPK-signaling activated by oncogenic BRAF^V600E^ and requires FRA-1, a AP-1 family member (24). Surprisingly, the suppression of SLUG in melanoma inhibits metastasis *in vivo* (28). This finding supports the idea that all EMT-inducing transcription factors promote malignant tumor progression rather than impeding it. In view of the clonal evolution model, Gupta et al. proposed that the paradoxical expression of SLUG in melanocytes and benign precursor lesions prior to transformation could limit the number of additional alterations required for clonal selection toward metastatic clones. In other words, the pre-existing expression of SLUG in melanocytes may predispose melanoma to its well-known metastatic potential. Although the hypothesis proposed by Gupta et al. seems to oppose the current view on the tumor-suppressive functions of SLUG in melanoma, their observations do not contradict the plasticity model described by Caramel et al.: the differentiation factor SLUG can allow reversion of the ZEB1/TWIST-driven invasive phenotype toward a differentiated phenotype that enables outgrowth of disseminated cancer cells similar to a mesenchymal-to-epithelial transition (MET) in carcinomas. The observation that SLUG can transcriptionally activate ZEB1 whereas ZEB1 is repressed by MITF suggests that a complex network of positive and negative feedback loops is regulating this reversible reprogramming (25, 29). Although EMT-inducing transcription factors may exert antagonizing effects during development and progression of melanoma, this antagonizing trade-off is likely the key to successful metastatic spread and colonization (Figure 1).

## Phenotype-Switching and Environmental Cues

Changes in the microenvironment are presumed to direct phenotype-switching (13). In line with this, phenotype-switching can be initially triggered by genetic instability that sensitizes the cells toward microenvironmental cues. Although the role of oncogene signaling in directing the reversible reprograming of EMT-inducing transcription factors in melanoma is well established (30), the microenvironmental signals that contribute to this reprograming are largely unknown. One major candidate signaling factor directing this reversible switching is TGF-β. The extensive interplay between Zeb transcription factors and members of the TGF-β family in normal and pathological context of epithelial cells is well established (3, 31). The findings suggest that varying levels of TGF-β could also directly regulate the expression of EMT-inducing transcription factors during melanoma phenotype-switching. TGF-β is critical in the regulation of Gli2, which in turns has antagonistic effect on MITF expression, eventually leading to melanoma invasion and metastasis (32, 33). Moreover, Gli2 cooperates with ZEB1 to transcriptionally repress of E-cadherin in melanoma, and TGF-β strongly enhances this complex formation (34). These data identify ZEB1 as a major player during the cadherin switch in melanoma. Whether the repression of MITF by ZEB1 described by Denecker et al. depends on TGF-β and a formation of the GLI2 complex formation is unknown. In comparison, TGF-β is a key mediator of normal melanocyte stem cell maintenance and quiescence (35). The detection of ZEB1 protein in melanocyte stem cells further implicates a TGF-β-ZEB1 circuitry relevant to both melanocyte and melanoma stemness (Figure 1) (25). One of the microenvironmental influences promoting the switch from a proliferative to an invasive phenotype is hypoxia. Key findings from the studies by Cheli et al. (36) and Widmer et al. (37) show that under hypoxic conditions MITF expression is downregulated in a HIF1α-dependent fashion while EMT-associated markers such as ZEB1, SNAIL, fibronectin (FN1), Sparc, and matrix metalloproteinase 2 (MMP2) are increased (36, 37). Moreover, hypoxic conditions increase the tumorigenic potential of melanoma cells whereas pharmacological depletion of a MITF-negative population by forskolin treatment inhibits tumor and metastasis development. In line with this, immunohistochemical analysis of melanomas showing signs of hypoxia identified a FN1^high^MITF^low^ subpopulation expressing presumed melanoma cancer-stem cell markers such as ABCB5, HIF2A, JARID1B, and NGFR as well as ZEB1 and SNAIL (38).

Reversible phenotypic plasticity of melanoma cells can also be driven by environmental inflammatory signals, e.g., it can be initiated by UV-damaged epidermal keratinocytes releasing HMGB1 and subsequent neutrophil infiltration (39). UV-dependent activation and recruitment of neutrophils facilitates angiogenesis and migration along a path defined by endothelial cells. Inflammation-induced phenotypic switching toward a dedifferentiated state may underlie the mechanism by which melanoma patients develop resistance to adoptive cell therapy (ACT) (40). ACT uses cytotoxic T cells to target melanoma-specific antigens, which are lost upon the reversible dedifferentiation driven by the inflammatory cytokine TNF-α. Importantly, the authors show that the switch is reversible, melanoma cells reacquire expression of the differentiation markers after treatment and cessation of inflammation (40). The emerging concept of phenotype-switching in melanoma points us to new therapeutic possibilities. Nevertheless, chemotherapy itself may provoke cancer cell populations to adopt a reversible drug-tolerant phenotype. Sharma et al. (41) reported that melanoma cells transiently maintain a drug-tolerant stage through chromatin modifications and active IGF-1R signaling (41). In a recent “differentiation therapy,” melanoma was made susceptible to specific drugs. After methotrexate-mediated upregulation of MITF in melanoma cells, tyrosinase expression was induced. Subsequently, the pro-drug TMECG was activated by tyrosinase and consequently inhibited dihydrofolate reductase (DHFR) leading to apoptosis of melanoma cells (42).

## Balancing Melanoma Heterogeneity

The cancer-stem cell model, the clonal evolution model, and the phenotype-switching model are not mutually exclusive, because clonal evolution is likely to precede cancer stemness. In addition, genetic instability in the tumor generates the necessary genetic variation, and consequently transcriptomic and proteomic diversity, which allow microenvironment-driven or cell-intrinsic phenotype-switching. Nevertheless, the relative contributions of these different sources of tumor heterogeneity may vary depending on tumor type. Melanoma cells are endowed with unique features of reversible cellular plasticity, they reversible switch between non-tumorigenic and tumorigenic state. So therefore the cancer-stem cell model might not effectively represent tumors possessing high interconversion rate. Therefore, phenotype-switching is increasingly appreciated as a mechanism accounting for melanoma heterogeneity. Indeed, the cancer-stem cell hypothesis that implies the existence of a rare stem cell fraction was challenged with the observation that 28% of single melanoma cells obtained from patients-derived tumors were tumorigenic in NOD/SCID IL2Rγ^null^ mice (43). Besides the suggested influence of technical and assay-dependent factors, the high frequency of tumor-initiating cells could also be partially attributed to the intrinsic plasticity of melanoma. Instead of the idea that a small subset of cells possessing cancer-stem cell characteristics, the phenotypic switching model implies that most cells can adopt an invasive stem cell-like-identity driven by microenvironmental cues. When melanoma cells are triggered by the appropriate microenvironmental signals, they can readily metastasize while bypassing the acquisition and selection process of pro-invasive mutations. A proliferative and differentiated phenotype can be reestablished by appropriate microenvironments, similar to MET in carcinomas (44). As described above, regaining MITF and SLUG expression is beneficial for the outgrowth of metastatic cells and may even be required for expansion of dormant cells at distant sites. Generation of an invasive cancer-stem cell-like subpopulation probably occurs in all tumor types, but the probably unique way by which it predominantly arises in melanoma exemplifies the extremely metastatic potential and cellular plasticity of melanoma compared to many other tumor types.

The physiological development of melanocytes is likely the key to the unique features of cellular heterogeneity and plasticity arising during the pathological development of melanoma. Molecular pathways active in the normal melanocyte differentiation program and consisting of MITF and EMT-inducing transcription factors may explain the ability of melanoma cells to easily switch toward an invasive phenotype. It should be noted that EMT itself does not exist in melanoma as melanocytes are not epithelial cells. EMT plays a key role during the formation and migration of neural crest cells. Neural crest cells are a multipotent, migratory, transient cell population that migrates through the vertebrate embryo to infiltrate different organs and differentiate in various cell lineages including melanocytes. Therefore, melanocytes can be regarded as a product of embryonic EMT.

## Conflict of Interest Statement

The authors declare that the research was conducted in the absence of any commercial or financial relationships that could be construed as a potential conflict of interest.
